# Regulation of temporal cytokine production by co-stimulation receptors in TCR-T cells is lost in CAR-T cells

**DOI:** 10.1093/immadv/ltae004

**Published:** 2024-06-19

**Authors:** Ashna Patel, Mikhail A Kutuzov, Michael L Dustin, P Anton van der Merwe, Omer Dushek

**Affiliations:** The Sir William Dunn School of Pathology, University of Oxford, Oxford OX1 3RE, UK; The Sir William Dunn School of Pathology, University of Oxford, Oxford OX1 3RE, UK; The Kennedy Institute of Rheumatology, University of Oxford, Oxford OX3 7FY, UK; The Sir William Dunn School of Pathology, University of Oxford, Oxford OX1 3RE, UK; The Sir William Dunn School of Pathology, University of Oxford, Oxford OX1 3RE, UK

**Keywords:** T cells, chimeric antigen receptors, T-cell receptors, co-stimulation receptors, cytokine production, immmunotherapy

## Abstract

CD8+ T cells contribute to immune responses by producing cytokines when their T-cell receptors (TCRs) recognise peptide antigens on major-histocompability-complex class I. However, excessive cytokine production can be harmful. For example, cytokine release syndrome is a common toxicity observed in treatments that activate T cells, including chimeric antigen receptor (CAR)-T-cell therapy. While the engagement of costimulatory receptors is well known to enhance cytokine production, we have limited knowledge of their ability to regulate the kinetics of cytokine production by CAR-T cells. Here we compare early (0–12 h) and late (12–20 h) production of IFN-gg, IL-2, and TNF-a production by T cells stimulated via TCR or CARs in the presence or absence ligands for CD2, LFA-1, CD28, CD27, and 4-1BB. For T cells expressing TCRs and 1st-generation CARs, activation by antigen alone was sufficient to stimulate early cytokine production, while co-stimulation by CD2 and 4-1BB was required to maintain late cytokine production. In contrast, T cells expressing 2nd-generation CARs, which have intrinsic costimulatory signalling motifs, produce high levels of cytokines in both early and late periods in the absence of costimulatory receptor ligands. Losing the requirement for costimulation for sustained cytokine production may contribute to the effectiveness and/or toxicity of 2nd-generation CAR-T-cell therapy.

## Graphical Abstract

**Graphical Abstract:**
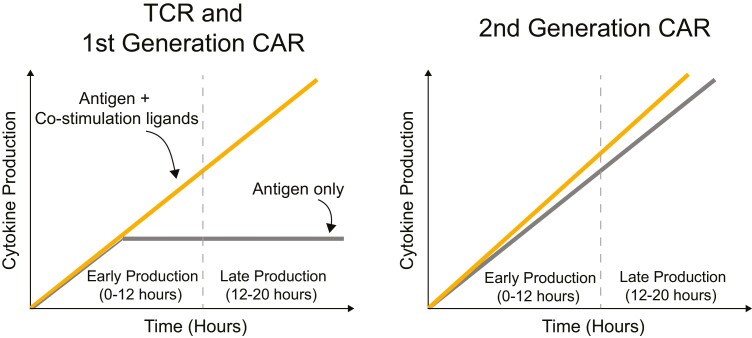

## Introduction

CD8^+^ T cells play central roles in immunity to intracellular pathogens and cancer. They can become activated to respond when their cell surface T-cell receptors (TCRs) recognise peptide antigens displayed on major-histocompatibility-complex (pMHC) class I gene products on the surface of nearly all cells. The binding of pMHC to the TCR initiates a signalling cascade that can lead to multiple functional responses. These include the production of cytokines such as IFN-g, IL-2, and TNF-*a* that collectively contribute to the ability of CD8^+^ T cells to execute immune responses [1, 2].

Because excessive cytokines levels can damage tissues, their production needs to be tightly regulated. One mechanism of regulation is engagement of co-stimulatory and/or co-inhibitory receptors on the T-cell surface, which increases and decreases the production of cytokines, respectively [3–6]. For example, cytokine production by T cells in cancer and chronic infections requires engagement of CD28 [7]. We have limited information on the relative abilities of other co-stimulatory receptors, such as the adhesion receptors CD2 and LFA-1 and the TNFSF receptors CD27 and 4-1BB, to sustain T-cell cytokine production.

The use of T cells in cell therapies to treat cancers, autoimmunity, and intractable chronic infections is showing great promise [8–10]. In these therapies, T cells are transduced to express a new TCR (TCR-T) or chimeric antigen receptor (CAR-T) that recognises an appropriate target antigen before being infused back into patients. While TCR-T and 1st-generation CAR-T lack intrinsic co-stimulatory activity, 2nd-generation CAR-T contains co-stimulatory signalling motifs in their cytoplasmic domains. The role of costimulatory receptors and intrinsic co-stimulatory signalling motifs in CAR-T-cell activation has previously been investigated [11–17] but their role in regulating sustained cytokine production by TCR-T and CAR-T cells remains poorly characterised. In the case of CAR-T cells, this is particularly important because patients often experience cytokine release syndrome, which can lead to organ failure and death [18].

**Figure 1.**
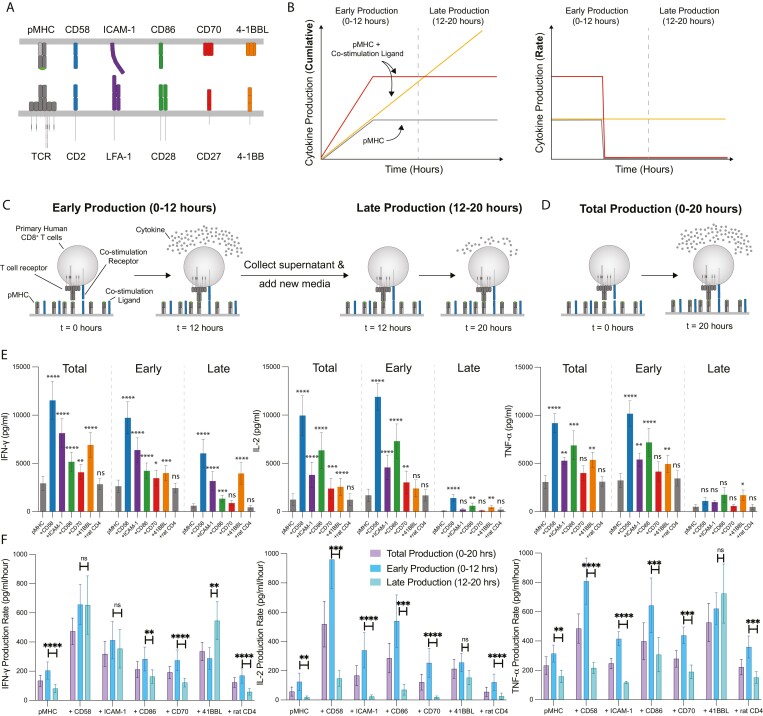
Regulated production of cytokine by co-stimulation receptors for the TCR. (A) Schematic of costimulation receptors and their ligands. (B) Schematics of potential cytokine production kinetics when T cells recognise antigen alone or with ligands to co-stimulation receptors. (C, D) Schematic of experiments to determine early, late, and total cytokine production. (E) Primary human CD8^+^ T cells transduced with the 1G4 TCR were stimulated by streptavidin surfaces coated with biotinylated pMHC alone (250 ng/well) or with the indicated biotinylated ligand (250 ng/well). The supernatant level of the indicated cytokine was determined after 20 h of stimulation (total production), after 12 h (early production), or between 12 and 20 h (late production). The rat CD4 ligand is used as a non-binding ligand control. Statistical significance of each ligand is compared to pMHC alone condition. (F) The production rate of each cytokine is calculated by dividing the supernatant cytokine data from (E) by the stimulation duration: 20, 12, or 8 h for total, early, or late, respectively. Statistical significance was determined by multiple *t*-tests on log-transformed data with Holm–Sidak correction for multiple comparisons. Abbreviations: *P-value < 0.05, **P-value < 0.01, ***P-value < 0.005, ****P-value < 0.001, ns = not significant. Data is shown as a mean ± SEM of six independent experiments (i.e. six independent human donors).

We have previously developed and validated a reductionist system using TCR/CAR and co-stimulatory receptor ligands, and used it to investigate antigen sensitivity of TCRs and CARs [15, 17, 19–22] (Fig. 1A). In this work, we use this system to investigate production of the cytokines IFN-g, IL-2, and TNF-*a* (Fig. 1B). We found that, while cytokine production by TCR-T and 1st generation CAR-T cells was dependent on engagement of co-stimulation receptors, cytokine production by 2nd generation CAR-T cells was largely independent of co-stimulation receptors.

## Materials and methods

### Protein production and purification

#### Ligands for co-stimulation receptors

The soluble extracellular domain (ECD) fused to a c-terminus His-tag and AviTag of human CD58, ICAM-1, CD86, 4-1BBL, and CD70 were produced in adherent HEK 293T cells. Plasmids encoding TNFSR ligands (4-1BBL and CD70) were a kind gift from Harald Wajant (Wurzburg, Germany) and incorporate a Flag¨ tag and tenascin-C trimerisation domain [50]. Adherent HEK 293T cells were transfected with Xtremegene HP reagent (Roche) according to manufacturer’s protocol. Supernatants were filtered using a 0.45 µM filter and proteins purified on a Ni-NTA agarose column. Biotinylation of proteins was performed either *in vitro* using the BirA enzyme (Avidity, USA) or *in situ* through co-transfection of BirA encoding plasmid (10%) with 100 µM D-biotin. Additional purification with removal of excess biotin was performed through size exclusion chromatography in HBS-EP. Purified proteins were stored at −80°C.

#### Peptide-MHC monomers

The 9V variant (SLLMWITQV) derived from the wild-type NY-ESO-1_157 165_ 9C peptide was synthesised at a purity of *>*95% (Peptide Protein Research, UK). Class 1 pMHCs were produced by expressing Human HLA-A*0201 heavy chain and human -2microglobulin with a C-terminal BirA tag in *Escherichia coli* as inclusion bodies and refolded with the 9V peptide *in vitro* as described previously [22, 24]. Protein was biotinylated using the BirA enzyme (Avidity, USA) and purified via size-exclusion chromatography (Superdex S75 column, GE Healthcare, USA), in HBS-EP buffer (10 mM M HEPES pH 7.4, 150 mM NaCl, 3 mM EDTA, 0.005% v/v Tween-20). Purified protein was aliquoted and stored at −80°C until use.

#### Peptide-MHC tetramers

Fluorescently conjugated pMHC tetramers were produced to enable the detection of antigen receptors (TCR/ CAR). Streptavidin-PE was gradually added to biotinylated 9V pMHC at a 4:1 molar ratio while shaking. Tetramers were stored for up to 3 months at 4°C in the dark.

### Production of TCR or CAR transduced primary human CD8+ T cells

#### Primary human CD8+ T-cell isolation

Human CD8+ T cells were isolated from leukocyte cones purchased from the National Health Service’s (UK) Blood and Transplantation service. Isolation was performed using negative selection. Briefly, blood samples were incubated with Rosette-Sep Human CD8+ enrichment cocktail (Stemcell) at 150 µl/ml for 20 min. This was followed by a 3.1-fold dilution with PBS before layering on Ficoll Paque Plus (GE) at a 0.8:1.0 ficoll to sample ratio. Ficoll-sample preparation was spun at 1200g for 20 min at room temperature. Buffy coats were collected, washed, and isolated cells counted. Cells were resuspended in complete RMPI (RPMI supplemented with 10% v/v FBS, 1% penicillin/ streptomycin) with 50 Units/ml of IL-2 (PeproTech) and CD3/CD28 human T-activator Dynabeads (Thermo Fisher) at a 1:1 bead to cell ratio. At all times isolated human CD8+ T cells were cultured at 37 and 5% CO_2_.

#### Lentivirus production

HEK 293T cells were seeded in DMEM supplemented with 10% FBS and 1% penicillin/streptomycin in six-well plates to reach 60–80% confluency on the following day. Cells were transfected with 0.25 pRSV-Rev (Addgene, 12253), 0.53 µg pMDLg/pRRE (Addgene, 12251), 0.35 µg pMD2.G (Addgene, 12259), and 0.8 µg of transfer plasmid using 5.8 X-tremeGENE HP (Roche). Media was replaced after 16 h and supernatant harvested after a further 24 h by filtering through a 0.45 cellulose acetate filter. Supernatant from one well of a six-well plate was used to transduce 1 million T cells.

#### TCR and CAR transduction

One million cells in 1 ml of complete RPMI were grown overnight in TC-treated 12 well plates. On the following day, T cells were transduced using lentivirus encoding for 1G4 TCR or CAR constructs. On Days 2 and 4 post-transduction, 1 ml of media was exchanged and IL-2 was added to a final concentration of 50 Units/ml. Dynabeads were magnetically removed on Day 5 post-transduction. T cells were then cultured at a density of 1 million cells/ml and supplemented with 50 Units/ml IL-2 every other day. T cells were used between 10 and 16 days after transduction.

#### Plate stimulation assay

Biotinylated pMHC and biotinylated costimulatory ligand (CD58, ICAM-1, CD86, CD70, or 4-1BBL) were diluted to the required concentration and added to Pierce Streptavidin Coated High Capacity 96 well plates (Thermo Fisher) in 50 µl PBS and incubated for 45 min at room temperature or left overnight at 4°C.

The 96-well plates were washed twice in PBS to remove excess protein. CD8^+^ T-cell blasts T cells transduced with TCR or CAR, were counted and washed before being added to wells in the 96 well plate. 75 000 T cells were added in 200 µl complete RPMI unless otherwise stated. Cells were spun briefly at 50g, 37°C for 1 min to allow them to settle at the bottom of each well and make contact with relevant protein, before incubation for the required time at 37°C 5% CO_2_. At the end of the stimulation assay, the supernatant was carefully removed and saved for ELISA analysis. 10 mM EDTA in PBS was then added to detach the T cells. The cells were then aspirated and transferred to a v-bottom plate and washed once in 200 µl PBS 1% BSA (500g, 4°C, 5 min). Antibodies against T-cell activation markers were diluted in PBS 1% BSA at a 1:200 dilution. To detect TCR-CAR expression fluorescently conjugated peptide-MHC tetramers were added to the staining antibodies at a 1:1000 dilution. A viability dye was also added at a dilution of 1:2500 to distinguish live cells from dead cells. The cells were treated with 50 µl of the staining solution before incubating them for 20 min at 4°C in the dark. The cells were washed twice in PBS, and resuspended in 75 µl PBS, before running on a flow cytometer. Flow cytometry data was analysed using FlowJo (BD Biosciences).

### Cytokine detection

IL-2 human uncoated ELISA kit, TNF-a human uncoated ELISA kit, IFN-g human uncoated ELISA kit, and Nunc MaxiSorp 96-well plates were used according to the manufacturer’s instructions. The supernatant from stimulation assays was diluted 1 in 15 for ELISAs. The absorbance at 450 nm and 570 nm were measured using a SpectraMax M5 plate reader (Molecular Devices).

### Data analysis

All statistical tests were performed in PRISM V.9 (GraphPad Software). ELISA standard curves were fitted using linear regression.

### CAR and TCR sequences

The CARs we have used have been previously described [17]. The 2nd generation CAR contains the following architecture:

D52N scFv—FLAG − CD28 hinge [P10747_112 152_] − CD28 TM [P10747_153 179_] − CD28 ICD [P10747_180 220_]. − CD247 *z*-chain ICD [P20963_52 164_] with UniProtKB accession numbers indicated and the D52N scFv sequence is

EVQLLESGGGLVQPGGSLRLSCAASGFTFSTYQMSWVRQAPGKGLEW

VSGIVSSGGSTAYADSVKGRFTISRDNSKNTLYLQMNSLRAEDTAVY

YCAGELLPYYGMDVWGQGTTVTVSSAKTTPKLEEGEFSEARVQSELT

QPRSVSGSPGQSVTISCTGTERDVGGYNYVSWYQQHPGKAPKLIIHN

VIERSSGVPDRFSGSKSGNTASLTISGLQAEDEADYYCWSFAGGYYV FGTGTDVTVLG

The 1st generation CAR is identical to the above except without the CD28 ICD.

The sequence of the 1G4 wild-type TCR we have used containing human constant domains is as follows

1G4 alpha-chain:

METLLGLLILWLQLQWVSSKQEVTQIPAALSVPEGENLVLNCS

FTDSAIYNLQWFRQDPGKGLTSLLLIQSSQREQTSGRLNASLD KSSGRSTLYIAASQPGDSATYLCAVRPTSGGSYIPTFGRGTSL

IVHPYIQNPDPAVYQLRDSKSSDKSVCLFTDFDSQTNVSQSKD

SDVYITDKTVLDMRSMDFKSNSAVAWSNKSDFACANAFNNSII

PEDTFFPSPESSCDVKLVEKSFETDTNLNFQNLSVIGFRILLL KVAGFNLLMTLRLWSS

1G4 beta-chain:

MSIGLLCCAALSLLWAGPVNAGVTQTPKFQVLKTGQSMTLQCA

QDMNHEYMSWYRQDPGMGLRLIHYSVGAGITDQGEVPNGYNVS RSTTEDFPLRLLSAAPSQTSVYFCASSYVGNTGELFFGEGSRL

TVLEDLKNVFPPEVAVFEPSEAEISHTQKATLVCLATGFYPDH

VELSWWVNGKEVHSGVSTDPQPLKEQPALNDSRYCLSSRLRVS

ATFWQNPRNHFRCQVQFYGLSENDEWTQDRAKPVTQIVSAEAW

GRADCGFTSESYQQGVLSATILYEILLGKATLYAVLVSALVLM

AMVKRKDSRG

## Results

### The temporal production of cytokines is tuned differently by different T-cell co-stimulation receptors

To study the impact of T-cell co-stimulation on the kinetics of cytokine production, we transduced primary human CD8^+^ T cells with the 1G4 TCR recognising a modified peptide from the NY-ESO-1_157 165_ cancer testis antigen (SLLMWITQV, 9V) displayed on HLA-A*02:01 [23, 24]. We stimulated these T cells with purified biotinylated pMHC antigen alone or together with the purified biotinylated extracellular domain of ligands to the co-stimulation receptors CD2 (CD58), LFA-1 (ICAM-1), CD28 (CD86), CD27 (CD70), and 4-1BB (4-1BBL) (Fig. 1A). We included purified biotinylated rat CD4 as a negative control protein to ensure that addition of ligands did not sterically impact the ability of T cells to recognise pMHC. We coupled biotinylated antigens and ligands to streptavidin surfaces because this stimulation platform allowed for precisely defined surfaces that could persistently activate T cells without other compensatory/redundant molecules. We have previously observed [15, 21] that co-stimulation can prevent T-cell desensitisation to TCR signals over a period of 20 h leading to different outcomes for cytokine accumulation and production rates (Fig. 1B). To parse cytokine production kinetics the amount of IFN-g, IL-2, and TNF-*a* was measured after 12 h of stimulation (early production), the media was then replaced and the amount of the same cytokines were measured over the next 8 h (late production) (Fig. 1C). As a control, we also measured the total cytokine produced after 20 h without replacing the media (total production) (Fig. 1D).

Consistent with their known costimulatory role, all tested costimulatory ligands increased total production of almost all cytokines (Fig. 1E). The single exception was that CD70 did not increase TNF-*a* production. The CD2 costimulatory ligand CD58 had the largest impact on the production of all three cytokines. The second most potent costimulatory ligand varied with cytokine: it was the LFA-1 ligand ICAM-1 for IFN-g and the CD28 ligand CD86 for IL-2 and TNF-*a*.

Interestingly, distinct effects were seen when comparing early versus late production (Fig. 1E), especially for IL-2 and TNF-*a*. While most ligands increased early production of each cytokine, only some increased late production. For example, CD2, LFA-1, CD28, and 4-1BB engagement increased early production of TNF-*a* but only 4-1BB engagement increased late production.

To directly compare early and late production, we calculated the cytokine production rate by dividing by the stimulation duration (Fig. 1F). The rate of cytokine production decreased between early and late production for all three cytokines when T cells were stimulated with pMHC antigen alone. Interestingly, co-stimulatory ligands differed in their effect on late cytokine production. 4-1BB co-stimulation maintained the production rate of IL-2 and TNF-a, and increased the production rate of IFN-g. CD2 and LFA-1 costimulation maintained the production rate of IFN-g, but not IL-2 and TNF-a. Finally, CD28 and CD27 co-stimulation failed to maintain the production rate of any of the three cytokines.

Previous work has suggested that T cells can tune their cytokine production based on feedback from extracellular cytokine levels [25, 26]. To exclude this we examine the effect of varying the density of T cells in the culture (Supplementary Fig. S1). Our finding that cytokine levels scaled linearly with cell numbers shows that cytokine production is not affected by extracellular cytokine levels, ruling out feedback effects in this experimental system.

Taken together, these results indicate that sustained (20 h) cytokine production induced by TCR engagement requires co-stimulation and that co-stimulatory receptors differ in their impact on cytokine production.

### Unregulated cytokine production by 2nd generation CAR-T cells

**Figure 2.**
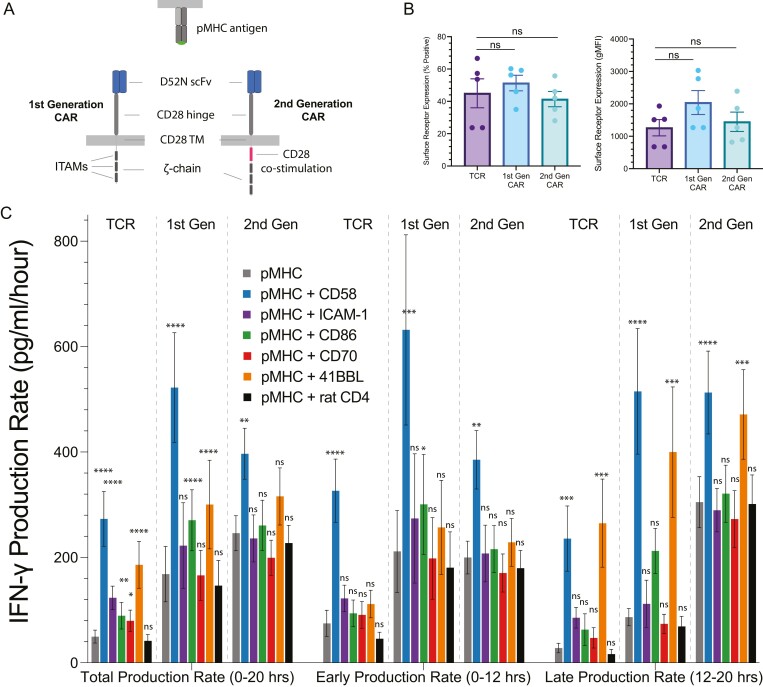
Regulated production of the cytokine IFN-g by co-stimulation receptors for 1st generation CAR-T cells is reduced for 2nd generation CAR-T cells. (A) Schematic of 1st and 2nd generation CARs that use the D52N scFv to recognize the NY-ESO-1 pMHC. (B) Surface expression of each antigen receptor on transduced primary human CD8^+^ T cells is detected using pMHC tetramers in flow cytometry. (C) Rate of IFN-g production for the indicated conditions. Statistical significance was determined by multiple *t*-test on log-transformed data relative to the pMHC alone condition with a Holm–Sidak multiple comparisons correction. Abbreviations: *P-value < 0.05, **P-value < 0.01, ***P-value < 0.001, ****P-value < 0.0001, ns = not significant. Data is shown as a mean ± SEM of five independent experiments (i.e. five independent human donors).

We next compared the early and late production of cytokines by CAR-T cells. We used a previously described 1st generation CAR containing the *z*-chain cytoplasmic domain and a 2nd generation CARs contain both the *z*-chain and CD28 cytoplasmic domains [17]. These CARs use the D52N scFv, which like the 1G4 TCR, recognise the NY-ESO-1 pMHC (Fig. 2A). The D52N scFv is derived from the 3M4EF Fab, which binds pMHC in the same orientation as the TCR [27, 28]. In previous work we found that these CARs required antigen for cytokine production and that CAR expression did not induce T-cell exhaustion, suggesting that these CARs produce little (or no) tonic signalling [17]. Using pMHC tetramers, we confirmed that both CARs expressed at similar levels to the TCR (Fig. 2B).

**Figure 3.**
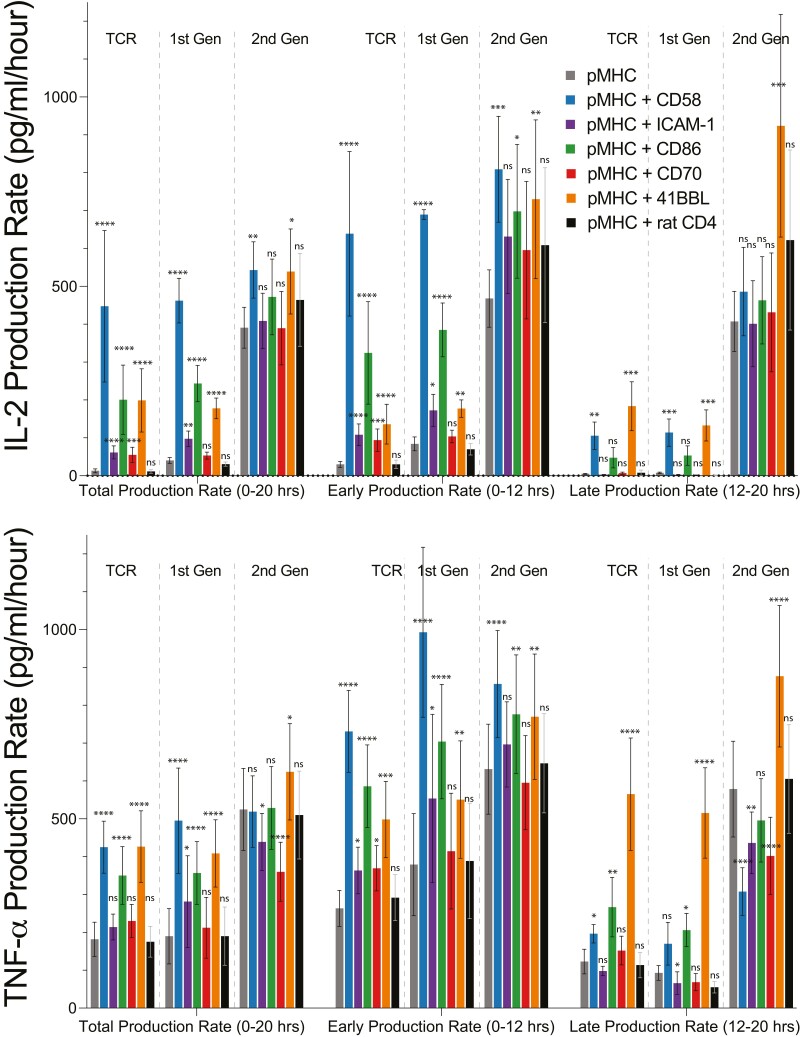
Regulated production of the cytokine IL-2 and TNF-a by co-stimulation receptors for 1st generation CAR-T cells is reduced for 2nd generation CAR-T cells. The production rate of IL-2 (top) and TNF-a (bottom) for the indicated antigen receptor, production period, and co-stimulation ligand. Statistical significance was determined by multiple *t*-test on log-transformed data relative to the pMHC alone condition with a Holm–Sidak multiple comparisons correction. Abbreviations: *P-value < 0.05, **P-value < 0.01, ***P-value < 0.001, ****P-value < 0.0001, ns = not significant. Data is shown as a mean ± SEM of five independent experiments (i.e. five independent human donors).

We measured total, early, and late cytokine production by TCR and CAR-expressing CD8^+^ T cells in response to pMHC antigen alone or with co-stimulation ligands. As before, we measured cytokine levels (Supplementary Fig. S2) and calculated production rates by dividing by the stimulation duration (Figs. 2C and 3). Only CD2 engagement increased early IFN-g production induced by CAR stimulation, while CD2 and 4-1BB engagement increased late IFN-g production (Fig. 2C). CD2, CD28, and 4-1BB engagement enhanced early IL-2 and TNF-a production induced by stimulation via TCR and both CARs (Fig. 3). CD2, CD28, and 4-1BB engagement also enhanced late IL-2 and TNF-a production induced by stimulation via TCR and the 1st generation CAR (Fig. 3). However, only 4-1BB engagement enhanced late IL-2 and TNF-a production induced by stimulation of the 2nd generation CAR (Fig. 3).

**Figure 4.**
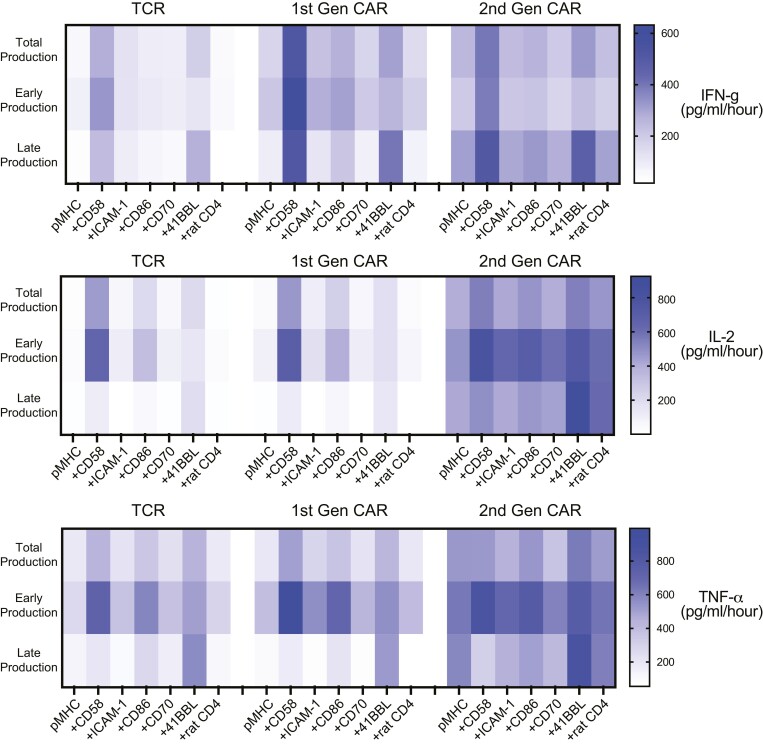
Cytokine production rates are maintained by 2nd generation CAR-T cells largely independent of extrinsic co-stimulation. The mean cytokine production rates from Figs. 2 and 3 are displayed in heatmaps.

Interestingly T cells expressing a 2nd generation CAR had a striking ability to maintain a high rate of late cytokine production in response to antigen alone (Figs. 2–3). This is more easily observed when plotting the data as heatmaps (Fig. 4) or when plotting early cytokine production against late cytokine production (Supplementary Fig. S3). In the case of the TCR and 1st generation CARs, the production rate of all cytokines decreased between early and late periods in response to antigen alone. Engagement of CD2 or 4-1BB was required to maintain late cytokine production. In striking contrast, 2nd generation CARs were able to maintain a high rate of cytokine production in response to antigen alone, even without co-stimulation.

As a control experiment, we examined whether the sum of early and late production was equal to the total production for each cytokine (Supplementary Fig. S4). We found that the sum of early and late exhibited a modest increase over total production for all three cytokines. A likely explanation for this is increased degradation/consumption of cytokines after secretion in the total versus the early/late production experiments. In the early/late experiments, supernatant is removed after 12 h and stored at low temperatures, which would halt degradation/consumption. An alternative explanation is that the secreted cytokine-induced negative feedback reduces cytokine production rates preferentially in the total stimulation experiment. However, we failed to find evidence for secreted cytokine levels influencing production rates in our cell titration experiments (Supplementary Fig. S1).

## Discussion

Using a reductionist system, we have found that cytokine production by T cells is highly regulated by extrinsic co-stimulation ligands when recognising antigens through the TCR or 1st generation CAR. However, 2nd generation CARs displayed largely unregulated cytokine production that is maintained independent of extrinsic co-stimulation receptors. The analysis we have performed can be extended in a number of ways, including the study of co-inhibitory receptors and combinations thereof.

The ability of T cells to orchestrate immune responses relies on finely tuned production of cytokines. This may be important to enable the clearance of pathogens without excessive tissue damage [7]. We found that the rate of cytokine production decreases when T cells recognise antigens in isolation, as in our previous work [15, 21]. As a result, T cells become increasingly reliant on signalling from co-stimulation receptors to sustain cytokine production. The sustained production of IFN-g, IL-2, and TNF-a over 20 h could be achieved by 4-1BB co-stimulation whereas co-stimulation by CD2 and LFA-1 could only sustain IFN-g production. Although CD28 and CD27 co-stimulation increased the early rate of cytokine production (except TNF-a for CD27), they could not sustain this higher rate with CD27 co-stimulation completely absent over the late production period. The impact of all of these co-stimulation receptors has previously been shown to be TCR dependent [15, 21, 29–32], although in the case of 4-1BB there is also evidence for a TCR independent signalling function [33, 34], which may explain its unique ability to sustain cytokine production. We have used expanded primary human CD8^+^ T cells and a similar ‘wave’ of cytokine production has been observed in a variety of un-expanded primary T cells, including naive, central memory, and effector memory when stimulated by the TCR and CD28 [35]. Taken together, these results suggest that sustained cytokine production by T cells relies on extrinsic co-stimulation receptors.

In contrast to the TCR, we found that antigen recognition by 2nd generation CARs produced largely unregulated cytokine production. Whereas the TCR and 1st generation CARs required extrinsic ligation of co-stimulation receptors to increase and sustain cytokine production, 2nd generation CARs maintained (IL-2, TNF-a) or increased (IFN-g) their rate of cytokine production over time independent of extrinsic co-stimulation. The only detectable regulation was by 4-1BB which led to an even higher production rate of all cytokines, which may be a result of the non-overlapping signalling pathways induced by CD28 and 4-1BB [3, 33]. We hypothesise that this regulation by the 4-1BB co-stimulation receptor will be lost in 2nd generation CARs that rely on the 4-1BB rather than the CD28 co-stimulation domain. Moreover, we predict that 3rd generation CARs that contain both CD28 and 4-1BB co-stimulation domains would not exhibit higher cytokine production by engagement of CD28 and 4-1BB receptors, as their signalling pathways would be fully activated by the CAR [36, 37].

The ability of co-stimulation receptors to increase cytokine production by a population of cells may result from increasing the fraction of activated T cells and/or increasing the amount of cytokine produced by each T cell [38–40]. However, this dichotomy can be blurred in situations where T cells can be activated (e.g. as measured by surface CD69) but where cytokine cannot be detected. In these situations, co-stimulation receptors may amplify intracellular signalling to engage cytokine production pathways in already activated T cells. Indeed, we have used a high concentration of pMHC in the present study (250 ng/well), which we previously determined activates the majority of T cells in this experimental system as determined by single-cell flow cytometry for surface CD69 [17]. T cells produce only modest levels of the cytokine IL-2 in this condition in the absence of co-stimulation. It follows that engaging co-stimulation receptors is stimulating IL-2 production from already activated T cells. Similarly, the early production of IFN-g induced by antigen alone is lost in late production for the TCR unless co-stimulation is included, suggesting that T cells were initially activated. Taken together, these results suggest that co-stimulation receptors act to induce cytokine production in already activated T cells.

Ligand engagement of co-stimulation receptors could enhance cytokine production in part by increasing cell adhesion. We note that CD70 and 4-1BBL are both high-affinity trimers [41] yet only 4-1BBL is able to sustain late cytokine production. Conversely, CD86, ICAM-1, and CD58 are lower-affinity monomers [42–44] yet CD58 generally has a larger impact in late production compared to CD86 and ICAM-1. Thus ligand binding strength/valency does not correlate with costimulatory activity. More likely explanations include differences in signalling pathways or expression patterns of costimulation receptors. In previous work, we have suggested that the TCR-signalling induced expression of 4-1BB but not CD27 can explain the ability of 4-1BB but not CD27 to sustain cytokine production [21].

A key difference between TCRs and CARs is their affinity for their target antigens. While TCRs typically bind their antigens with *K*_D_ values larger than 1 µM [45], antibody-derived CARs bind antigens with *K*_D_ values lower than 1 µM and often into the nM regime [46, 47]. Our finding that 1st generation CAR displayed a similar pattern of regulated cytokine production to the TCR, despite binding with a 50-fold higher affinity [17], suggests that differences in antigen affinity do not explain the difference reported in this study between the TCR and 2nd generation CARs. The CAR/antigen affinity has been shown to control antigen sensitivity [48, 49], including for the D52N CAR used in this study [17].

The unregulated production of cytokines by CAR-T cells is a double-edged sword. On the one hand, cancer cells are known to reduce the expression of ligands to co-stimulation receptors to escape T-cell immunity. As a result, reducing the dependence on co-stimulation ligands for cytokine production is a useful feature in reducing the ability of cancers to evade CAR-T cell responses. On the other hand, the inability to extrinsically control CAR-T cells means that the normal regulatory processes of adaptive immunity are lost increasing the possibility of cytokine storms leading to excessive inflammatory responses and tissue damage.

## Supplementary Material

ltae004_suppl_Supplementary_Material

## Data Availability

The data underlying this article are available in the article and in its online supplementary material.

## Abbreviations

CAR: Chimeric antigen receptor
CRS: Cytokine release syndrome;
pMHC: peptide major-histocompatibilitycomplex
TCR: T cell receptor
TNFSF: Tumour necrosis factor superfamily
