# Supplementary material for: Regulation of temporal cytokine production by co-stimulation receptors in TCR-T cells is lost in CAR-T cells
Source: Immunother Adv. 2024 Jun 19;4(1):ltae004. doi: 10.1093/immadv/ltae004 (PMC11228853; doi:10.1093/immadv/ltae004)
Supplement: ltae004_suppl_Supplementary_Material [file ltae004_suppl_supplementary_material.pdf]

Supplementary Information
Regulation of temporal cytokine production by co-stimulation receptors in TCR-T cells is lost in CAR-T cells

Ashna Patel[1], Mikhail A. Kutuzov[1],
Michael L. Dustin[2], P. Anton van der Merwe[1], Omer Dushek[1,¶]

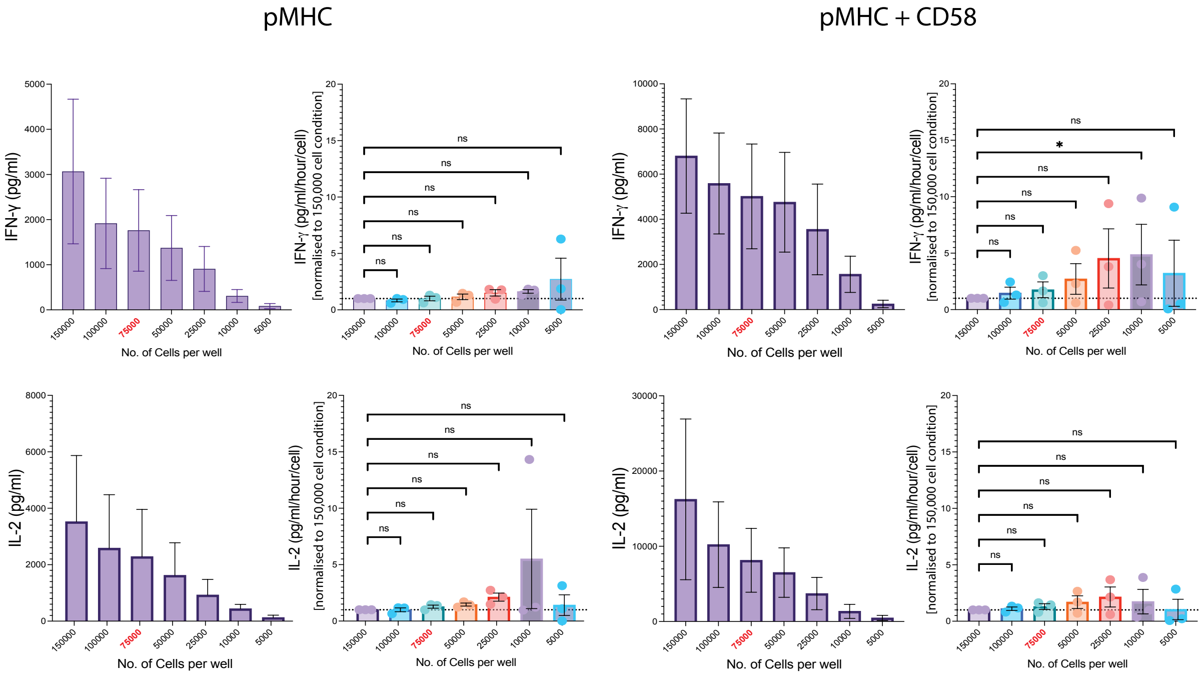

Figure S1: **Cytokine production scales linearly with T cell number.** The indicated number of primary human CD8^{+} T cells transduced with the 1G4 TCR (from 150,000 per well to 5000 per well) were stimulated by pMHC or by pMHC and CD58 for 20 hours before measuring IFN-γ (top row) or IL-2 (bottom row). All other experiments in the manuscript were performed with 75,000 cells per well (indicated in red). Statistical significance was determined by 1-way ANOVA on log-transformed data. Abbreviations: * = p-value$\leqslant$0.05.

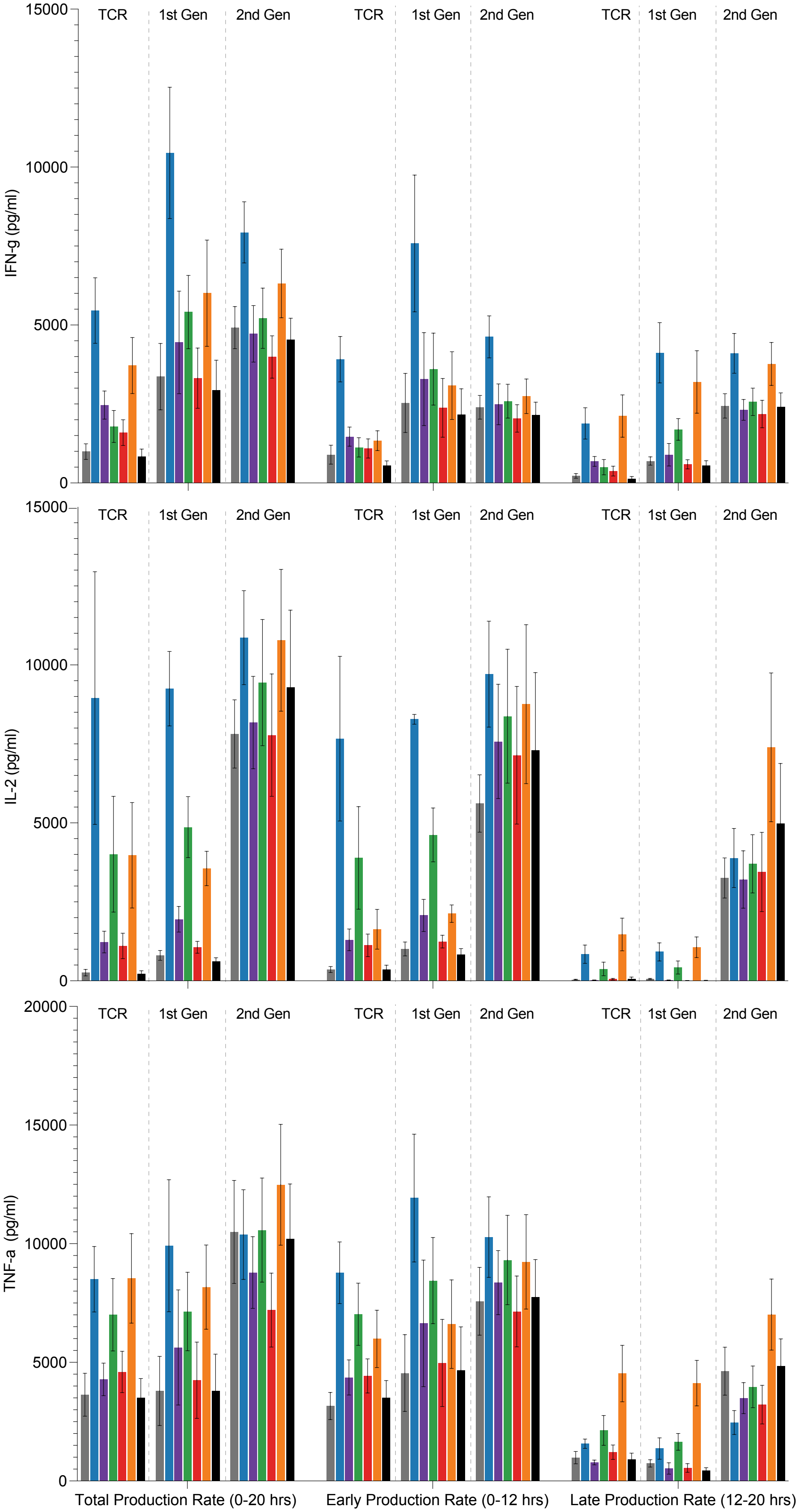

Figure S2: **The absolute cytokine levels from Fig 2-3 without normalisation for the stimulation period.**

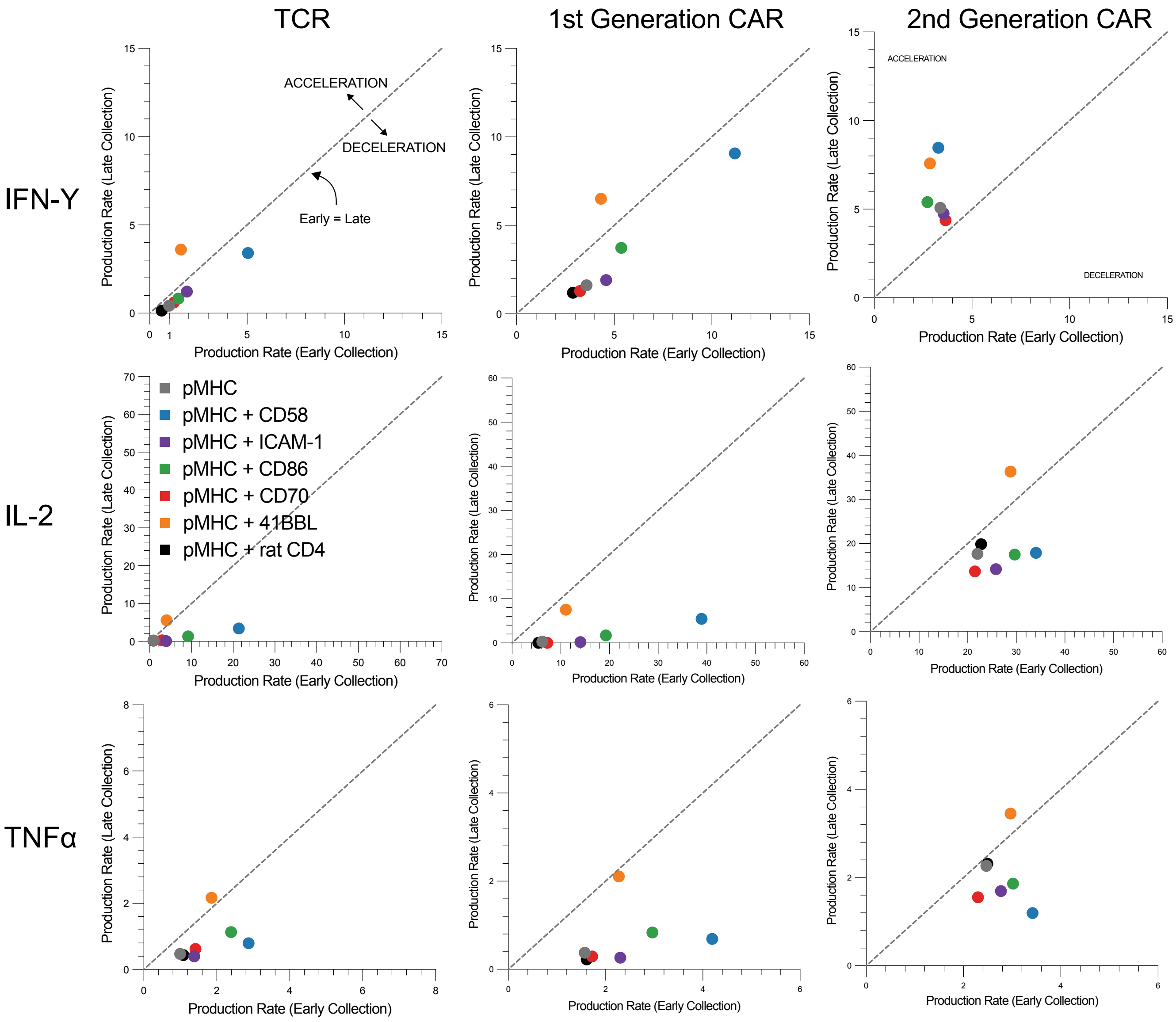

Figure S3: **Cytokine production rates are maintained by 2nd generation CAR-T cells largely independent of extrinsic co-stimulation.** The late production rate is plotted over the early production rate for the indicated cytokine (rows) and antigen receptor (columns). All data is replotted from Fig. 4 normalised to the early rate of cytokine production by the TCR in response to pMHC alone. All statistical analysis can be found in Fig. 2-3. Data is shown as a mean of 5 independent experiments (i.e. 5 independent human donors).

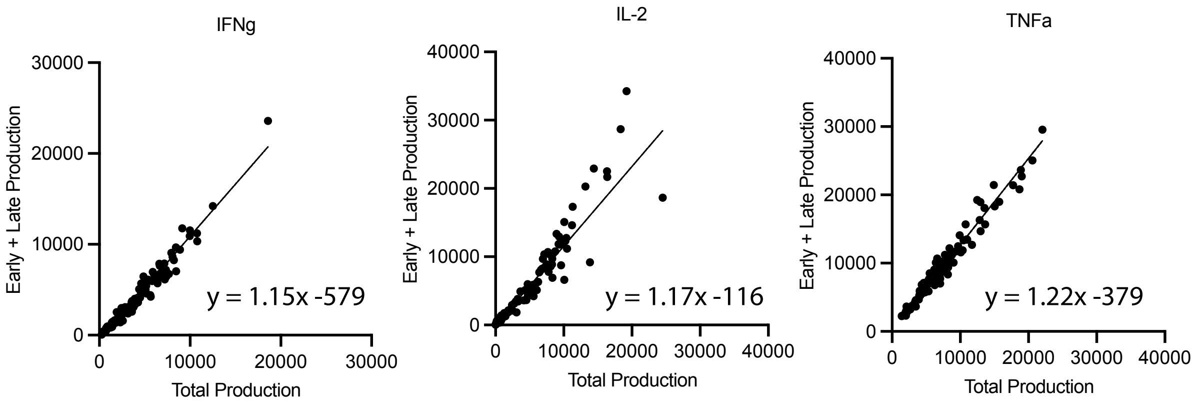

Figure S4: **The total cytokine production is similar to the sum of early and late cytokine production**. The sum of the absolute cytokine levels from Fig. S2 for early and late production is plotted over total production for all antigen receptors and all stimulation conditions.
